# Fasting confers stress resistance to skeletal muscle stem cells through non-metabolic actions of β-hydroxybutyrate: implications in cardioprotection and aging

**DOI:** 10.20517/jca.2022.24

**Published:** 2022-07-04

**Authors:** Junichi Sadoshima

**Affiliations:** Department of Cell Biology and Molecular Medicine, Rutgers New Jersey Medical School, Newark, NJ 07103, USA

## Abstract

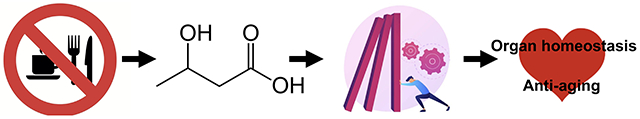

Dietary caloric restriction (CR) and fasting generally induce cell-protective effects in various organisms ranging from yeast to mammals^[1]^. They also consistently promote lifespan and healthspan in various organisms. Although they often affect common signaling mechanisms, including sirtuins and mTOR, and cellular mechanisms, such as autophagy, how they affect upstream mechanisms, such as metabolism and substrate utilization, to control cellular homeostasis, regeneration, and aging, is not fully understood. Among the major nutrients, namely glucose, protein, and fat, fat is the most prominently mobilized and used in the presence of starvation^[2]^. In particular, the utilization of ketone bodies, water-soluble derivatives of fat that readily penetrate the blood-brain barrier, replaces glucose oxidation in the brain in response to fasting, which in turn spares gluconeogenesis and preserves body proteins. Increasing lines of evidence suggest that ketone bodies play an important role in mediating the salutary effects of CR and fasting^[3]^.

In a recent paper published in *Cell Metabolism*, Benjamin *et al.* demonstrated that fasting delays muscle regeneration acutely but promotes a state of deep quiescence (DQ) characterized by downregulation of cell proliferation genes and upregulation of genes involved in stemness in muscle stem cells (MuSCs)^[4]^. MuSCs with DQ are highly stress-resistant and exhibit improved survival during stress. Importantly, the effect of fasting is mediated by ketosis, which can be mimicked by ketogenic diet consumption or exogenously administered β-hydroxybutyrate (BHB). BHB directly promotes MuSC DQ independently of its metabolism, namely through inhibition of HDAC1 and acetylation/activation of p53 [Figure 1].

The study is highly significant because it provides novel insights regarding the mechanism through which fasting confers stress resistance to MuSCs. The study also clearly demonstrates the non-metabolic role of BHB as a signaling molecule mediating the effect of fasting. Furthermore, the authors demonstrated that BHB causes MuSCs to enter the highly protective DQ state, improving their self-renewal over the long term. Although the study describes the effect of fasting upon skeletal muscle regeneration, several aspects of the findings are relevant to the cardioprotective effect of fasting.

The study by Benjamin *et al.* showed that fasting confers a high level of resilience to MuSCs against stress by inducing the DQ state^[4]^. MuSCs also exhibit less myogenic but more *stem-like* properties in response to fasting. These properties would allow skeletal muscle to maintain its regenerative capacity for a long period of time. It is uncertain whether resident stem cells are present in the heart and how they may contribute to mediating myocardial regeneration compared to the proliferation of existing cardiomyocytes^[5]^. Thus, it remains unclear whether the salutary effect of fasting in the heart is mediated through cardiac stem cells or individual cardiomyocytes. However, if fasting, BHB, and HDAC1 inhibition confer resilience to cardiac resident cells, these interventions may assist in identifying a resident stem cell population in the heart. Furthermore, since a low engraftment capacity remains a major obstacle during cell therapy with mesenchymal stem cells^[6]^, fasting, BHB, and HDAC1 inhibition before injection may improve the efficiency of cell therapy by conferring resilience. Even if the post-natal regenerative capacity of the heart by cardiac stem cells is negligible, fasting may confer stress resistance to existing cardiomyocytes by activating similar signaling mechanisms.

The importance of BHB in mediating the salutary actions of fasting and CR has been demonstrated in mammals and the multiple organs therein^[3]^. BHB generally plays an adaptive role in the heart, not only as an alternative fuel during heart failure but also as a signaling molecule that directly inhibits HDAC1 and promotes downstream cell protective mechanisms, including upregulation of thioredoxin 1 and suppression of mTOR^[7–10]^. Thus, the study by Benjamin *et al.* provides a strong rationale for testing whether the salutary effect of fasting is mediated by BHB in the heart as well and, if so, exploring small molecules that mimic the action of BHB or interventions that induce ketosis as CR mimetics^[4]^. Furthermore, interventions that induce ketosis can be used as a surrogate for a chemical inhibitor of HDAC1^[11]^, a cardioprotective intervention that inhibits cardiac remodeling^[12]^. Benjamin *et al.* showed that p53 acetylation is a major effector of HDAC1 inhibition and mediates fasting-induced DQ in MuSCs^[4]^. Since p53 generally promotes detrimental effects in the heart, other targets of HDAC1, including FoxO3a, whose acetylation is increased during fasting, could be explored as distal mediators of stress resistance in the heart^[11]^. It should be noted that HDAC1 inhibition due to glycosphingolipid imbalance in aging hearts leads to an aberrant elevation in histone acetylation and promotes DNA damage and aging^[13]^. Thus, the consequence of HDAC1 inhibition in the heart requires careful assessment.

Although the study by Benjamin *et al.* provides many insights, several important questions remain. DQ is defined by a delayed entry into the S phase of the cell cycle and enhanced resilience to nutrient, cytotoxic, and proliferative stress^[4,14]^. MuSCs in DQ also exhibit a significant reduction in mitochondrial content, RNA content, and basal oxygen consumption. In addition, they exhibit a unique transcriptomic profile, namely an increased ratio of p21 to cyclin D1, lower expression of myogenic genes, and increased expression of CD34, a MuSC stemness marker. Although MuSCs in DQ express cell cycle inhibitors, including p21 and p19, and display resilience to stress, both of which are the major features of cellular senescence, these MuSCs also display properties distinct from those of senescent cells since the cell size is smaller and p16 upregulation is not observed^[4]^. It has been shown that the level of lysosomal activity determines the depth of quiescence: decreases in lysosomal function promote the depth of quiescence and the ultimate establishment of cellular senescence^[14]^. Although Benjamin *et al.* stated that MuSCs subjected to ketosis exhibit a transcriptomic profile similar to that of DQ fibroblasts in response to altered lysosomal activity *in vitro*, whether the lysosomal activity is decreased or how lysosomal activity affects the depth of quiescence is not addressed in MuSCs^[4]^. Since the salutary action of BHB may be mediated by stimulation of autophagy^[15]^, the relationship between the status of lysosomal function and the induction of DQ needs to be scrutinized in MuSCs. Furthermore, what exactly distinguishes DQ from senescence needs to be clarified, given that they mediate diametrically opposite cellular functions. If DQ and senescence are part of a continuum^[14]^, a better understanding of the mechanisms driving DQ and senescence may lead to the development of interventions that stimulate DQ while inhibiting cellular senescence.

CR is one of the most established interventions to extend lifespan and healthspan in mammals^[1]^. Furthermore, many mechanisms extending lifespan in lower organisms confer stress resistance to the organism and the organs therein, which is the basis of the hormesis hypothesis^[16]^. Thus, the ultimate question is whether interventions to increase BHB and/or inhibition of HDAC1 also prevent aging in the heart and, if so, whether the effect upon cardiac aging is mediated by the DQ status in cardiac stem cells or a DQ-equivalent mechanism in cardiomyocytes themselves. Since some adverse effects of a long-term ketogenic diet and HDAC1 inhibition have been noted^[13,17]^, however, caution should be exercised when evaluating the efficacy of these interventions.

In summary, the study by Benjamin *et al.* showed that fasting confers resilience to MuSCs by promoting development of the DQ state^[4]^. Although the presence of equivalent resident stem cells in the heart and the significance of the DQ-like status in cardiac cells remain uncertain, it appears that similar mechanisms, including BHB and HDAC1 inhibition, are activated by fasting and play a protective role in the heart. Thus, further investigation of the downstream mechanism by which BHB and HDAC1 inhibition protect the heart against stress may provide useful information to protect the heart against myocardial injury, prevent aging, and promote healthspan in the heart.

## Figures and Tables

**Figure 1.**
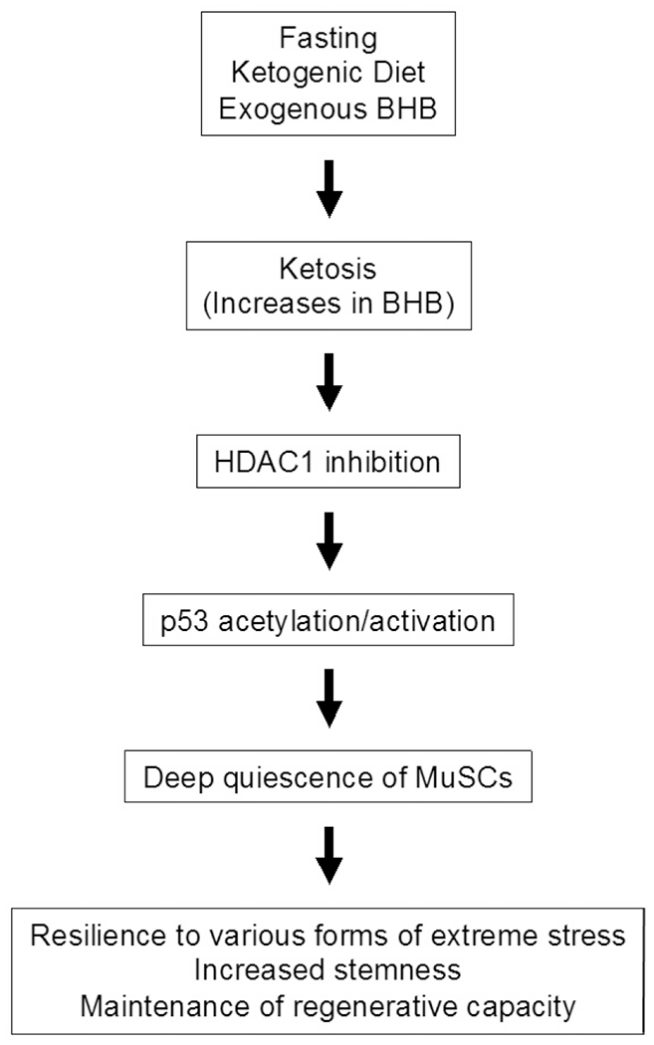
A schematic representation of the induction of stress resistance by fasting in skeletal muscle stem cells. The scheme summarizes the finding of Benjamin *et al.*^[4]^. BHB: β-hydroxybutyrate; HDAC1: histone deacetylase 1; MuSCs: muscle stem cells.

## Data Availability

Not applicable.
